# Phase I/II study of weekly irinotecan and concurrent radiation therapy for locally advanced non-small cell lung cancer

**DOI:** 10.1038/sj.bjc.6690233

**Published:** 1999-03

**Authors:** K Takeda, S Negoro, S Kudoh, K Okishio, N Masuda, M Takada, M Tanaka, T Nakajima, T Tada, M Fukuoka

**Affiliations:** Department of Pulmonary Medicine, Department of Radiology,Osaka City General Hospital, 2-13-22, Osaka, Miyakojima-ku, 534-0021, Japan; First Department of Internal Medicine, First Department of Radiology, Osaka City University School of Medicine, 1-5-7, Osaka, Abeno-ku, 545-0051, Japan; Second Department of Internal Medicine,Department of Radiology, Osaka Prefectural Habikino Hospital, 3-7-1, Osaka, Habikino, 583-0872, Japan; Fourth Department of Internal Medicine, Kinki University, School of Medicine, Osaka-Sayama, Ohnohigashi, 589-0014, Japan

**Keywords:** irinotecan, chemoradiotherapy, clinical trial, radiosensitization

## Abstract

A study was undertaken to determine the maximum tolerated dose, the dose-limiting toxicities, and the response rate of irinotecan administered weekly with concurrent thoracic radiation therapy in patients with locally advanced non-small-cell lung cancer. In a phase I/II clinical trial, patients with histologically documented, surgically unresectable stage IIIA or IIIB non-small cell lung cancer (NSCLC) were enrolled. Irinotecan was administered as a 90 min intravenous infusion once weekly for 6 weeks. The starting dose was 30 mg m^−2^ and dose escalation was done in 15 mg m^−2^ increments. Dose-limiting toxicity was defined as grade 3 nonhaematologic toxicity (excluding nausea, vomiting and alopecia) or grade 4 haematologic toxicity according to the WHO criteria. Radiation was delivered to the primary tumour and regional lymph nodes (40 Gy), followed by a boost to the primary tumour (20 Gy). Twenty-seven patients were entered into this study at three irinotecan dose levels (30, 45 and 60 mg m^−2^). Twenty-six eligible patients were evaluated for toxic effects and clinical outcome. Severe oesophagitis, pneumonitis, and diarrhoea occurred at 45 and 60 mg m^−2^. Three of the five patients given 60 mg m^−2^ developed grade 3 or 4 oesophagitis and pneumonitis. In addition, one patient died of pneumonitis after completing therapy at 45 mg m^−2^ in the phase II study. The objective response rate was 76.9% (95% CI, 53.0–88.9%). Oesophagitis, pneumonitis, and diarrhoea are the dose-limiting toxicities of weekly irinotecan combined with thoracic irradiation. The maximum tolerated dose and the dose for the phase II study were 60 and 45 mg m^−2^ wk^−1^, respectively. This combined therapy for locally advanced non-small cell lung cancer is promising and shows acceptable toxicity. © 1999 Cancer Research Campaign


British Journal of Cancer (1999) **79**(9/10), 1462–1467
© 1999 Cancer Research Campaign
Article no. bjoc.1998.0233

### Phase I/II study of weekly irinotecan and concurrent radiation therapy for locally advanced non-small cell lung cancer

**K Takeda[1], S Negoro[1], S Kudoh[3], K Okishio[3], N Masuda[5], M Takada[5], M Tanaka[2], T Nakajima[4], T Tada[6] and M Fukuoka[7]**

Departments of [1]Pulmonary Medicine and [2]Radiology, Osaka City General Hospital, 2-13-22, Miyakojimahondori, Miyakojima-ku, Osaka 534-0021, Japan; [3]First Department of Internal Medicine and [4]Radiology, Osaka City University School of Medicine, 1-5-7, Asahicho, Abeno-ku, Osaka 545-0051, Japan; [5]Second Department of Internal Medicine and [6]Department of Radiology, Osaka Prefectural Habikino Hospital, 3-7-1, Habikino, Habikino, Osaka 583-0872, Japan; [7]Fourth Department of Internal Medicine, Kinki University, School of Medicine, 377-2, Ohnohigashi, Osaka-Sayama 589-0014, Japan

**Summary** A study was undertaken to determine the maximum tolerated dose, the dose-limiting toxicities, and the response rate of irinotecan administered weekly with concurrent thoracic radiation therapy in patients with locally advanced non-small-cell lung cancer. In a phase I/II clinical trial, patients with histologically documented, surgically unresectable stage IIIA or IIIB non-small cell lung cancer (NSCLC) were enrolled. Irinotecan was administered as a 90 min intravenous infusion once weekly for 6 weeks. The starting dose was 30 mg $m^{-2}$ and dose escalation was done in 15 mg $m^{-2}$ increments. Dose-limiting toxicity was defined as grade 3 nonhaematologic toxicity (excluding nausea, vomiting and alopecia) or grade 4 haematologic toxicity according to the WHO criteria. Radiation was delivered to the primary tumour and regional lymph nodes (40 Gy), followed by a boost to the primary tumour (20 Gy). Twenty-seven patients were entered into this study at three irinotecan dose levels (30, 45 and 60 mg $m^{-2}$). Twenty-six eligible patients were evaluated for toxic effects and clinical outcome. Severe oesophagitis, pneumonitis, and diarrhoea occurred at 45 and 60 mg $m^{-2}$. Three of the five patients given 60 mg $m^{-2}$ developed grade 3 or 4 oesophagitis and pneumonitis. In addition, one patient died of pneumonitis after completing therapy at 45 mg $m^{-2}$ in the phase II study. The objective response rate was 76.9% (95% CI, 53.0–88.9%). Oesophagitis, pneumonitis, and diarrhoea are the dose-limiting toxicities of weekly irinotecan combined with thoracic irradiation. The maximum tolerated dose and the dose for the phase II study were 60 and 45 mg $m^{-2}$ $wk^{-1}$, respectively. This combined therapy for locally advanced non-small cell lung cancer is promising and shows acceptable toxicity.

**Keywords:** irinotecan; chemoradiotherapy; clinical trial; radiosensitization

Lung cancer is a leading cause of cancer death in many industrialized countries, with a 5-year survival rate of 14% at best (Wingo et al, 1995). Non-small cell lung cancer (NSCLC) accounts for approximately 75% of all lung cancer, and surgery offers the best chance of cure and long-term survival if the tumour is confined to the lung and is resectable. Unfortunately, the majority of patients present with disease not amenable to surgery because it either is locally advanced or has metastasized. For the approximately 25–30% of NSCLC patients who present with locally advanced cancer (stage IIIA or IIIB), fractionated thoracic radiation therapy has been the mainstay of treatment (Ihde et al, 1991). Despite such treatment, however, the overall outcome is invariably poor, with a median survival time that ranges from 9–13 months, while the 2- and 5-year survival rates are respectively 15–20% and 5–9% at best (Roswit et al, 1968; Holsti et al, 1980; Petrovich et al, 1981; Perez et al, 1982). Measures to improve the survival of these patients have been the subject of intense clinical investigation during the past two decades, with recent efforts being focused on multimodal therapy (Friess et al, 1987; Mattson et al, 1988; Dillman et al, 1990; Trovo et al, 1990; Le Chevalier et al, 1991; Morton et al, 1991; Sause et al, 1992; Schaake-Koning, 1992; Trovo et al, 1992; Sause et al, 1995; Jeremic et al, 1996).

*Received 19 May 1998*
*Revised 18 September 1998*
*Accepted 14 October 1998*

*Correspondence to:* K Takeda

Combined thoracic radiotherapy and chemotherapy for locally advanced NSCLC is theoretically appealing because it addresses the need to control the primary lesion while also attempting to eradicate occult distant micrometastases. Although the optimal sequencing of chemotherapy and radiotherapy is still unclear, most trials have used sequential rather than concurrent therapy, largely to avoid the anticipated greater toxicity with the latter approach.

Some anticancer drugs also act as radiation-sensitizing agents. For example, cisplatin is known to be a radiosensitizer (Douple, 1988), and when given in combination with radiotherapy to patients with inoperable NSCLC, it has been reported to improve both survival and local disease control at the price of causing substantial side-effects (Schaake-Koning, 1992).

Irinotecan is a derivative of camptothecin with a strong activity against NSCLC (Negoro et al, 1991). A phase II study of irinotecan for previously untreated NSCLC showed a high response rate of 31.9% (95% CI, 20.2–43.6%) (Fukuoka et al, 1992). A recent study of the combination of irinotecan with cisplatin showed a very promising response rate of 52% (95% CI,

39–64%) in previously untreated NSCLC patients with acceptable toxicities (Masuda et al, 1998).

Irinotecan is a potent topoisomerase I inhibitor that has undergone extensive clinical evaluation (Andoh et al, 1987). This agent is a prodrug with limited activity itself, which is converted by carboxylesterases into a biologically active metabolite (SN38) (Kawato et al, 1991). We showed that SN38 enhanced the radiosensitivity of a lung cancer cell line in vitro (Okishio et al, 1996). In addition, Tamura et al demonstrated that irinotecan combined with radiation significantly prolonged the survival time when compared with irinotecan or radiation alone in a small cell lung cancer xenograft model (Tamura et al, 1997).

Based on these reports of in vitro and in vivo radiation enhancement by irinotecan, we initiated a phase I/II trial of this drug combined with concurrent radiation therapy for locally advanced NSCLC. The major goals of the present study were: to determine the maximum tolerated dose of irinotecan administered as a 90 min weekly infusion along with daily thoracic radiation therapy in patients with locally advanced NSCLC, to determine the toxicities of combined irinotecan-radiation therapy, and to evaluate the response rate and feasibility of this regimen.

### PATIENTS AND METHODS

### Patient selection

Patients with histologically documented, surgically unresectable stage IIIA or IIIB NSCLC according to the criteria reported by Mountain (1986) were enrolled in this study. However, patients who had received previous chemotherapy or radiation therapy were excluded. A complete history and physical examination were performed in all patients. The nature and purpose of the study were fully explained to each patient. All patients signed an informed consent approved by the institutional review boards of Osaka City General Hospital, Osaka City University, School of Medicine, or Osaka Prefectural Habikino Hospital.

Patients were required to have measurable disease, an Eastern Cooperative Oncology Group (ECOG) performance status ≤ 2, an age ≤ 75 years, and no active concomitant malignancy. Patients with malignant pleural effusion were excluded. Measurable disease meant that the tumour was demonstrated by conventional chest roentgenograms or computed tomography (CT) of the chest. In addition, all patients underwent a routine staging evaluation that consisted of standard radiologic studies (including CT of the abdomen and brain) as well as bone scanning.

Eligibility requirements also included the following: white blood cell (WBC) count ≥ 4000 $mm^{-3}$, platelet count ≥ 100 000 $mm^{-3}$, haemoglobin ≥ 9.5 g $dl^{-1}$, serum bilirubin < 1.5 mg $dl^{-1}$, serum AST/ALT ≤ twice the upper limit of normal, serum creatinine less than the upper limit of normal, and arterial partial pressure of oxygen ($P\text{aO}_2$) ≥ 70 mmHg. Patients with markedly impaired pulmonary function (%VC < 70%, %TLC < 70% or $\%DL_{co}$ < 60%), and those with disease that required irradiation of more than half of the hemithorax were excluded from this study.

Height, weight, performance status, and tumour stage were recorded. Initial laboratory data obtained included a complete blood count, differential WBC count, platelet count, total and direct bilirubin, AST, ALT, alkaline phosphatase, total protein, albumin, blood urea nitrogen (BUN), creatinine, uric acid, serum electrolytes, calcium, phosphate, and $P\text{aO}_2$.

### Irinotecan dosage

Irinotecan was administered as a 90 min i.v. infusion once every week for 6 weeks. It was given at the start of the week before radiation therapy. Because the patients also received daily radiation therapy, the starting dose of irinotecan was only 30 mg $m^{-2}$. Three patients with NSCLC who required radiation therapy to the primary tumour site were entered at each dose level, and the dose was escalated in increments of 15 mg $m^{-2}$ for successive groups of three new patients until dose-limiting toxicity was observed. Dose-limiting toxicity was defined as grade 3 or 4 nonhaematologic toxicity excluding nausea, vomiting and alopecia or grade 4 haematologic toxicity according to the WHO toxicity criteria (World Health Organization, 1979). If one instance of dose-limiting nonhaematologic and/or haematologic toxicity was observed among three patients, an additional three patients were scheduled to be treated at the same dose level, and dose escalation would continue if dose-limiting toxicity was observed in only one or two out of six patients. When three instances of dose-limiting toxicity were observed among six patients, the present dose level was defined as the maximum tolerated dose (MTD). The dose for the phase II portion of the study was set one level lower than the MTD.

### Radiation therapy

Radiation therapy was performed concurrently with weekly irinotecan infusion for 6 weeks. The treatment volume consisted of original and boost volumes irradiated sequentially. The original volume included the primary disease site with a margin of 1.5 cm and the ipsilateral hilum. The entire width of the mediastinum was included, with a margin of 1.5 cm around the radiographically visible area of involvement on pretreatment chest X-ray films and CT scans. The ipsilateral supraclavicular fossa was treated from the cricoid cartilage laterally to the midclavicular line. The subcarinal lymph nodes were included to 5 cm below the carina. The boost volume included the original tumour volume and all lymph nodes greater than 2 cm in diameter visualized on CT scan with a margin of 1.5 cm.

The radiation dose to the original volume was 40 Gy in 20 fractions of 2.0 Gy over a period of 4 weeks, while the dose to the boost volume was 20 Gy in 10 fractions of 2.0 Gy over a period of 2 weeks. The spinal cord dose was limited to 40 Gy. Figure 1 summarizes the treatment schedule used in this study.

### Evaluation of response and toxicity

For the assessment of response and toxicity, the following tests were done once a week during treatment: complete blood count, AST, ALT, alkaline phosphatase, lactate dehydrogenase, bilirubin, creatinine, BUN, serum electrolytes, urinalysis, $P\text{aO}_2$, and chest X-ray film.

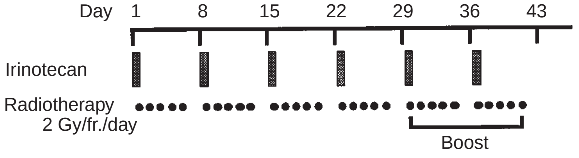

**Figure 1** Schedule for concurrent chemoradiotherapy

Response and toxicity were evaluated in accordance with WHO criteria (World Health Organization, 1979), except that grading of oesophageal toxicity due to radiation was done according to the ECOG criteria (Oken et al, 1982).

The eligibility, assessability, and response of each patient were determined by extramural review. The commissioned reviewer was expert in this area. A complete response was defined as the disappearance of all lesions for at least 4 weeks. A partial response was defined as a > 50% decrease in the sum of the products of the greatest perpendicular diameters of all measurable lesions for at least 4 weeks, without the development of new lesions. If no changes of the disease occurred during treatment, the patient was considered to have stable disease. Progressive disease was defined as a > 25% increase in the sum of the products of the perpendicular diameters of all measurable lesions, or the appearance of new lesions.

Differences in response rate between groups of patients were compared using chi-square ($\chi^2$) test. Survival was calculated on the basis of the period from the start of treatment to death or the last follow-up evaluation. Survival curves were drawn using the Kaplan–Meier method (Kaplan et al, 1958). Differences in survival estimates between groups of patients were evaluated using the log-rank test (Peto et al, 1977). All *P*-values were two-tailed.

**Table 1** Patient characteristics

| Characteristic | *n* | % |
|---|---|---|
| Enrolled | 27 | |
| Assessable | 26 | |
| Age (years) | | |
| Median | 63 | |
| Range | 32–75 | |
| Sex | | |
| Male | 20 | 76.9 |
| Female | 6 | 23.1 |
| Performance status (ECOG) | | |
| 0 | 1 | 3.8 |
| 1 | 21 | 80.8 |
| 2 | 4 | 15.4 |
| Weight loss within last 3 months | | |
| < 5% | 22 | 84.6 |
| ≥ 5% | 4 | 15.4 |
| Histology | | |
| Squamous cell carcinoma | 16 | 61.5 |
| Adenocarcinoma | 8 | 30.8 |
| Others | 2 | 7.7 |
| Stage | | |
| IIIA | 1 | 3.8 |
| IIIB | 25 | 96.2 |

### RESULTS

### Patients characteristics

Twenty-seven patients entered into this study through three dose escalations, and one patient was found to be ineligible because of metastatic disease. The main clinical characteristics of the 26 eligible patients are listed in Table 1. There were 20 male and six female, with a median age of 63 years (range: 32–75 years). Twenty-two patients (84.6%) had an ECOG performance status of 0 or 1. Each of four patients (15.4%) with more than 5% weight loss within the last 3 months also had an ECOG performance status of 2. Sixteen patients (61.5%) had squamous cell carcinoma, eight (30.8%) had adenocarcinoma, and two (7.7%) had other nonclassifiable types of NSCLC. One patient (3.8%) was in stage IIIA and 25 (96.2%) had stage IIIB disease, including one patient with recurrence after curative surgery.

Twelve of the 26 patients (46.2%) completed chemotherapy as scheduled. The major reasons for not completing the scheduled therapy were toxicity (10/14, 71.4%), cerebral infarction as an accidental complication (2/14, 14.3%), disease progression (1/14, 7.1%), and patient refusal (1/14, 7.1%). Eighteen of 26 patients (69.2%) completed radiation therapy according to the protocol, four (15.4%) completed it with minor variations, and four (15.4%) failed to complete it (two due to pulmonary toxicity and one each due to disease progression and cerebral infarction).

### Toxicity

Oesophagitis, pulmonary toxicity (pneumonitis), and diarrhoea were the dose-limiting toxicities of combined irinotecan-radiation therapy (Table 2). Grade 3 oesophagitis and diarrhoea occurred in one patient each at the 45 mg m$^{-2}$ dose level, but when three more patients received irinotecan at 45 mg m$^{-2}$, there were no further severe toxicities. At the 60 mg m$^{-2}$ level, grade 4 pneumonitis and grade 3 oesophagitis occurred in one patient each and one more patient developed grade 4 oesophagitis when treatment was extended at this dose level. Since three out of five patients developed severe toxicity, the maximum tolerated dose of irinotecan was scored as 60 mg m$^{-2}$ weekly and the dose used for the phase II study was 45 mg m$^{-2}$ weekly.

**Table 2** Major nonhaematologic toxicity

| | | | Toxicity (WHO grade) | | | | | | | | | | | | | | |
|---|---|---|---|---|---|---|---|---|---|---|---|---|---|---|---|---|---|
| | | | Oesophagitis | | | | | Pneumonitis | | | | | Diarrhoea | | | | |
| | Dose (mg m$^{-2}$) | Patients (*n*) | 0 | 1 | 2 | 3 | 4 | 0 | 1 | 2 | 3 | 4 | 0 | 1 | 2 | 3 | 4 |
| Phase I | 30 | 4 | 1 | 3 | 0 | 0 | 0 | 3 | 0 | 1 | 0 | 0 | 1 | 2 | 1 | 0 | 0 |
| | 45 | 7 | 0 | 3 | 3 | 1 | 0 | 6 | 1 | 0 | 0 | 0 | 4 | 0 | 2 | 1 | 0 |
| | 60 | 5 | 0 | 2 | 1 | 1 | 1 | 2 | 0 | 0 | 2 | 1 | 4 | 1 | 0 | 0 | 0 |
| Phase II | 45 | 10 | 3 | 4 | 3 | 0 | 0 | 4 | 1 | 4 | 0 | 1* | 7 | 2 | 1 | 1 | 0 |

*Treatment-related death

 *© Cancer Research Campaign 1999*

**Table 3** Haematologic toxicity

| | Dose (mg m$^{-2}$) | Patients (*n*) | WBC Count ($\times 10^3\ \mu l^{-1}$) | | Haemoglobin (g dl$^{-1}$) | | Platelet count ($\times 10^3\ \mu l^{-1}$) | |
|---|---|---|---|---|---|---|---|---|
| | | | Mean | Range | Mean | Range | Mean | Range |
| Phase I | 30 | 4 | 3.6 | 1.8–5.1 | 10.4 | 9.6–11.4 | 208 | 146–283 |
| | 45 | 7 | 3.4 | 2.0–7.7 | 10.1 | 8.5–11.8 | 242 | 89–379 |
| | 60 | 5 | 3.1 | 1.5–5.5 | 9.8 | 8.7–10.7 | 308 | 184–545 |
| Phase II | 45 | 10 | 2.6 | 1.6–5.9 | 10.1 | 8.7–11.4 | 198 | 118–350 |

**Table 4** Response to treatment

| | Dose level of CPT-11 (mg m$^{-2}$) | No. of pts | Response | | | | | Response rate (%) |
|---|---|---|---|---|---|---|---|---|
| | | | CR | PR | NC | PD | NE | |
| | 30 | 4 | 1 | 2 | 1 | 0 | 0 | 75.0 |
| Phase I | 45 | 7 | 0 | 4 | 2 | 0 | 1 | 57.1 |
| | 60 | 5 | 1 | 3 | 1 | 0 | 0 | 80.0 |
| Phase II | 45 | 10 | 1 | 8 | 0 | 0 | 1 | 90.0 |
| | Overall | 26 | 3 | 17 | 4 | 0 | 2 | 76.9* |

*95%CI 53.0–88.9%. CR: complete response; PR: partial response; NC: no change; PD: progressive disease; NE; not evaluable case

Ten patients were enrolled in the phase II study and two of them developed severe toxicity (grade 4 pneumonitis and grade 3 diarrhoea). The patient who developed Grade 4 pneumonitis died, and this was a treatment-related death. He completed chemoradiation therapy according to schedule, but developed a high fever and dyspnoea at the end of treatment. Chest X-ray films showed bilateral interstitial infiltrates. Oxygen and steroid pulse therapy were given, followed by intubation and mechanical ventilation, but he failed to respond.

Other nonhaematologic toxicities included dermatitis and nausea/vomiting. One patient developed grade 3 wet desquamation due to an allergic reaction to irinotecan and/or radiation at the 30 mg m$^{-2}$ dose level. Severe nausea or vomiting (grade 2 and 3) due to irinotecan occurred in eight out of 26 patients. 5-HT$_3$ antagonists were given prophylactically to patients with severe nausea or vomiting before the next infusion of irinotecan.

In contrast to the nonhaematologic toxicities, haematologic toxicity was mild at any dose level (Table 3).

No consisting or late toxicity was observed in this study.

### Response

The response to combined irinotecan-radiation therapy is shown in Table 4. Of the 26 patients, three achieved a complete response, 17 had a partial response, and two had stable disease. Two patients were not evaluable for response, including one who only received a single intravenous infusion of irinotecan and one who died of disease progression early in the treatment period. Overall, the objective response rate was 76.9% (95% CI: 53.0–88.9%). No significant differences in response were observed between the three dose levels of irinotecan.

### Survival

After a minimum follow-up period of 22 months, 16 patients have died (13 of documented disease progression and three of other diseases) and 10 patients remain alive at the time of analysis (five with and five without disease progression). No patient has been monitored for more than 36 months. The estimated 1- and 2-year survival rates were 61.5% and 38.5%, and the median survival time was 15.7 months for all eligible patients (Figure 2). Survival was also compared between the three dose levels of irinotecan. The 1-year survival rate was 50.0% at 30 mg m$^{-2}$, 64.7% at 45 mg m$^{-2}$, and 40.0% at 60 mg m$^{-2}$. There was no significant difference of survival in relation to the irinotecan dose.

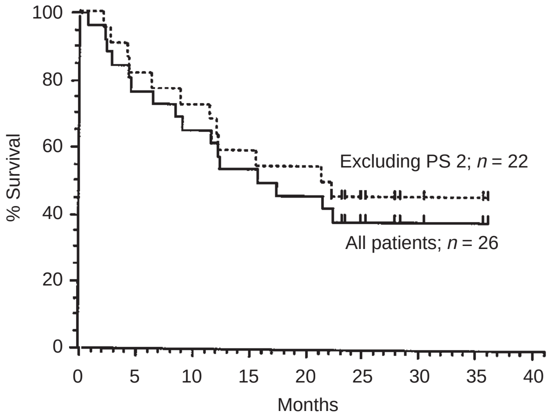

**Figure 2** Survival. The estimated 1- and 2-year survival rates were 61.5% and 36.0%, and the median survival time was 15.7 months for 26 eligible patients. Excluding patients with ECOG performance status of 2, the estimated 1- and 2-year survival rates were 68.2% and 45.5%, and the median survival time was 21.5 months

© Cancer Research Campaign 1999 

The estimated 1- and 2-year survival rates were 68.2% and 45.5%, and the median survival time was 21.5 months for the group of patients who had an ECOG performance status of 0 or 1, and less than 5% weight loss within the last 3 months.

### Pattern of failure

The sites of initial failure are shown in Table 5. The primary tumour inside the radiation field was the site of initial failure in eight patients (seven without and one with distant metastasis), while distant metastasis was the cause of failure in five patients. In five patients, including all three patients who achieved a complete response, there is no evidence of recurrent disease.

The estimated local progression-free survival rate was 49.0% at 1 year and 39.2% at 2 years, with a median of 11.6 months. The overall progression-free survival rate was 38.5% at 1 year and 30.8% at 2 years, with a median of 10.9 months.

### DISCUSSION

Several randomized trials comparing thoracic radiotherapy alone with radiotherapy plus chemotherapy have been reported. The first large trial to demonstrate a significant survival benefit with multimodal therapy was performed by the Cancer and Leukemia Group B (CALGB) (Dillman et al, 1990). Subsequently, the Radiation Therapy Oncology Group (RTOG) and the Eastern Cooperative Oncology Group (ECOG) performed a confirmatory trial (Sause et al, 1995). Both median and long-term survival were superior in the patients receiving multimodal treatment, confirming the results of the CALGB study. Favourable results have also been reported by French investigators (Le Chevalier et al, 1991). The overall incidence of distant metastasis was reduced in the chemotherapy-treated population, but local control was poor in both groups. These observations indicate that the survival benefit derived from chemotherapy comes from a reduction in the incidence of distant metastases rather than the radiation-sensitizing effect of the agents employed. The extremely poor local control rate is discouraging and suggests that further efforts to improve the control of the primary lesion are needed. The European Organization for Research and Treatment of Cancer (EORTC) performed a randomized study of inoperable NSCLC that compared split course radiotherapy alone versus the same radiotherapy plus cisplatin, administered daily or weekly (Schaake-Koning et al, 1992). Their findings confirmed the observation that cisplatin increases the therapeutic ratio of radiation, with the magnitude of the synergism depending on the administration schedule for the two agents. In addition, survival benefit of daily combined treatment was due to improved local control.

We initiated a first clinical trial of irinotecan combined with concurrent radiation therapy for locally advanced NSCLC in an attempt to increase locoregional control by employing its radiosensitizing effect. The present phase I/II study demonstrated that concurrent radiation therapy can be safely delivered with irinotecan at a dose of 45 mg $m^{-2}$ over 6 weeks in patients with locally advanced NSCLC. Oesophagitis, pneumonitis, and diarrhoea were the dose-limiting toxicities. Since oesophagitis and pneumonitis were severe at the highest dose level (60 mg $m^{-2}$ weekly for 6 weeks), 60 mg $m^{-2}$ was concluded to be the MTD and 45 mg $m^{-2}$ was the dose used in the phase II study. Although oesophagitis and pneumonitis were generally not so severe in the phase II study, one patient died of pneumonitis. This patient was a 71-year-old man with PS 1. His pretreatment profiles met the eligibility requirements in this study. However, he had a large tumour in the right lower lung and the radiation field was approximately half of the hemithorax. Thus, his fatal pneumonitis could have been related to the relatively large radiation field. Patients with large radiation field therefore have to be excluded in this combined therapy.

The objective response rate in the current study was 76.9% (95% CI, 53.0–88.9%). The median survival time was 15.7 months for all 26 patients, and the 1- and 2-year survival rates were 61.5% and 38.5%, respectively. Although the number of subjects is small in this study, these results compare favourably with those of other chemoradiation trials (Dillman et al, 1990; Le Chevalier et al, 1991; Morton et al, 1991; Sause et al, 1992; Schaake-Koning, 1992; Trovo et al, 1992; Sause et al, 1995; Jeremic et al, 1996).

The selection criteria employed by CALGB were fairly restrictive, so only patients with low-bulk disease (i.e. supraclavicular nodal involvement excluded), a good performance status (0 or 1), and minimal weight loss (5% or less of body weight) were included (Dillman et al, 1990). Thus, extrapolating the results of these trials to all stage III patients is potentially problematic. The RTOG and ECOG trial was done using virtually identical selection criteria, and it confirmed the results of the CALGB study (Sause et al, 1995).

In the present study, survival was at least as good as or better than that in the CALGB or RTOG-ECOG studies, although our entry criteria were less restrictive. The estimated 1- and 2-year survival rates were 68.2% and 45.5%, and the median survival time was 21.5 months for the group of patients excluding an ECOG performance status of 2, and more than 5% weight loss within the last 3 months. Compared with the reports by EORTC, our results were encouraging in survival benefits. Weekly administration of irinotecan combined with radiotherapy is sufficiently encouraging to merit further evaluation of the regimen in a randomized trial.

In conclusion, oesophagitis, pneumonitis, and diarrhoea were the dose-limiting toxicities of weekly irinotecan combined with thoracic irradiation. The MTD and the recommended dose were 60 mg $m^{-2}$ $wk^{-1}$ and 45 mg $m^{-2}$ $wk^{-1}$, respectively. This combined

**Table 5** Pattern of failure

| Site of initial failure | Patients (*n*) | % |
|---|---|---|
| Inside radiation field | 8 | 40 |
| Primary tumour site | 8 | 40 |
| Other site | 0 | 0 |
| Outside radiation field | 5 | 25 |
| Brain | 2 | 10 |
| Peritoneal lymph nodes | 2 | 10 |
| Contralateral supraclavicular lymph nodes | 1 | 5 |
| Response continued | 5 | 25 |
| Unknown* | 2 | 10 |

*Patients died without disease progression, including one who had treatment-related death, one who died of other disease

© Cancer Research Campaign 1999

therapy for locally advanced NSCLC appears to be promising and tolerable.

### ACKNOWLEDGEMENTS

We wish to thank Nobuhide Takifuji, Kazuhiko Terakawa, Takashi Nitta, and Tomonori Hirashima for their assistance in data monitoring. We also express our deep appreciation to Dr Shinichiro Nakamura for his extramural review. This work was supported in part by grants-in-aid from the Ministry of Health and Welfare (for the 2nd Term Comprehensive 10-Year Strategy for Cancer Control), the Ministry of Education, Science, Sports and Culture, Japan.

### REFERENCES

Andoh T, Ishii K, Suzuki Y, Ikegami Y, Kusunoki Y, Takemoto Y and Okada K (1987) Characterization of a mammalian mutant with a camptothecin-resistant DNA topoisomerase I. *Proc Natl Acad Sci USA* **84**: 5565–5569

Dillman RO, Seagren SL, Propert KJ, Guerra J, Eaton WL, Perry MC, Carey RW, Frei EF 3rd and Green MR (1990) A randomized trial of induction chemotherapy plus high-dose radiation versus radiation alone in stage III non-small-cell lung cancer. *N Engl J Med* **323**: 940–945

Douple EB (1988) Platinum-radiation interactions. In *NCI monographs, No. 6,* pp 315–319. Government Printing Office: Washington, DC

Friess GG, Baikadi M and Harvey WH (1987) Concurrent cisplatin and etoposide with radiotherapy in locally advanced non-small cell lung cancer. *Cancer Treat Rep* **71**: 681–684

Fukuoka M, Niitani H, Suzuki A, Motomiya M, Hasegawa K, Nishiwaki Y, Kuriyama T, Ariyoshi Y, Negoro S, Masuda N, Nakajima, S and Taguchi T (1992) A phase II study of CPT-11, a new derivative of camptothecin, for previously untreated non-small-cell lung cancer. *J Clin Oncol* **10**: 16–20

Holsti LR and Mattson K (1980) A randomized study of split-course radiotherapy of lung cancer: long term results. *Int J Radiat Oncol Biol Phys* **6**: 977–981

Ihde DC and Minna JD (1991) Non-small cell lung cancer. Part II: Treatment. *Curr Probl Cancer* **15**: 105–154

Jeremic B, Shibamoto Y, Acimovic L and Milisavljevic S (1996) Hyperfractionated radiation therapy with or without concurrent low-dose daily carboplatin/etoposide for stage III non-small-cell lung cancer: a randomized study. *J Clin Oncol* **14**: 1065–1070

Kaplan EL and Meier P (1958) Nonparametric estimation from incomplete observations. *J Am Stat Assoc* **53**: 457–481

Kawato Y, Aonuma M, Hirota Y, Kuga H and Sato K (1991) Intracellular roles of SN-38, a metabolite of the camptothecin derivative CPT-11, in the antitumor effect of CPT-II. *Cancer Res* **51**: 4187–4191

Le Chevalier T, Arriagada R, Quoix E, Ruffie P, Martin M, Tarayre M, Lacombe-Terrier MJ, Douillard JY and Laplanche A (1991) Radiotherapy alone versus combined chemotherapy and radiotherapy in nonresectable non-small cell lung cancer: first analysis of a randomized trial in 353 patients. *J Natl Cancer Inst* **83**: 417–423

Masuda N, Fukuoka M, Fujita A, Kurita Y, Tsuchiya S, Nagao K, Negoro S, Nishikawa H, Katakami N, Nakagawa K and Niitani H (1998) A phase II trial of combination of CPT-11 and cisplatin for advanced non-small-cell lung cancer. CPT-11 Lung Cancer Study Group. *Br J Cancer* **78**: 251–256

Mattson K, Holsti LR, Holsti P, Jakobsson M, Kajanti M, Liippo K, Mantyla M, Niitamo-Korhonen S, Nikkanen V, Nordman E, Platin LH, Pyrhonen S, Romppanen ML, Salmi R, Tammilehto L and Taskinen PJ (1988) Inoperable non-small cell lung cancer: radiation with or without chemotherapy. *European Journal of Cancer and Clinical Oncology* **24**: 477–482

Morton RF, Jett JR, McGinnis WL, Earle JD, Therneau TM, Krook JE, Elliott TE, Mailliard JA, Nelimark RA, Maksymiuk AW, Drummond RG, Laurie JA, Kugler JW and Anderson RT (1991) Thoracic radiation therapy alone compared with combined chemoradiotherapy for locally unresectable non-small cell lung cancer. A randomized, phase III trial. *Ann Intern Med* **115**: 681–686

Mountain CF (1986) A new international staging system for lung cancer. *Chest* **89**: 225S–233S

Negoro S, Fukuoka M, Masuda N, Takada M, Kusunoki Y, Matsui K, Takifuji N, Kudoh S, Niitani H and Taguchi T (1991) Phase I study of weekly intravenous infusions of CPT-11, a new derivative of camptothecin, in the treatment of advanced non-small-cell lung cancer. *J Natl Cancer Inst* **83**: 1164–1168

Oken MM, Creech RH, Tormey DC, Horton J, Davis TE, McFadden ET and Carbone PP (1982) Toxicity and response criteria of the Eastern Cooperative Oncology Group. *Am J Clin Oncol* **5**: 649–655

Okishio K, Kudoh S, Kurihara N, Hirata K and Takeda T (1996) Irinotecan (CPT-11) enhances the radiosensitivity of lung cancer cells in vitro. *Cellular Pharmacology* **3**: 247–252

Perez CA, Stanley K, Grundy G, Hanson W, Rubin P, Kramer S, Brady LW, Marks JE, Prez-Tamayo R, Brown GS, Concannon JP and Rotman M (1982) Impact of irradiation technique and tumor extent in tumor control and survival of patients with unresectable non-oat cell carcinoma of the lung. *Cancer* **50**: 1091–1099

Peto R, Pike MC, Armitage P, Breslow NE, Cox DR, Howard SV, Mantel N, McPherson K, Peto J and Smith PG (1977) Design and analysis of randomized clinical trials requiring prolonged observation of each patient. II. Analysis and examples. *Br J Cancer* **35**: 1–39

Petrovich Z, Stanley K, Cox JD and Paig C (1981) Radiotherapy in the management of locally advanced lung cancer of all cell types. Final report of randomized trial. *Cancer* **48**: 1335–1340

Roswit B, Patno ME, Rapp R, Veinbergs A, Feder B, Stuhlbarg J and Reid CB (1968) The survival of patients with inoperable lung cancer: a large-scale randomized study of radiation therapy versus placebo. *Radiology* **90**: 688–697

Sause WT, Scott C, Taylor S, Byhardt RW, Banker FL, Thomson JW, Jones TK, Cooper JS and Lindberg RD (1992) Phase II trial of combination chemotherapy and irradiation in non-small cell lung cancer: Radiation Therapy Oncology Group 88–04. *Am J Clin Oncol* **15**: 163–167

Sause WT, Scott C, Taylor S, Johnson D, Livingston R, Komaki R, Emami B, Curran WJ, Byhardt RW, Turrisi AT, Dar AR and Cox JD (1995) Radiation Therapy Oncology Group (RTOG) 88-08 and Eastern Cooperative Oncology Group (ECOG) 4588: preliminary results of a phase III trial in regionally advanced, unresectable non-small-cell lung cancer. *J Natl Cancer Inst* **87**: 198–205

Schaake-Koning C, van den Bogaert W, Dalesio O, Festen J, Hoogenhout J, van Houtte P, Kirkpatrick A, Koolen M, Maat B, Nijs A, Renaud A, Rodrigus P, Schuster-Uitterhoeve L, Sculier JP, van Zandwijk N and Bartelink H (1992) Effects of concomitant cisplatin and radiotherapy on inoperable non-small-cell lung cancer. *N Engl J Med* **326**: 524–530

Tamura K, Takada M, Kawase I, Tada T, Kudoh S, Okishio K, Fukuoka, M, Yamaoka N, Fujiwara Y and Yamakido M (1997) Enhancement of tumor radio-response by irinotecan in human lung tumor xenografts. *Jpn J Cancer Res* **88**: 218–223

Trovo MG, Minatel E, Veronesi A, Roncadin M, De Paoli A, Franchin G, Magri DM, Tirelli U, Carbone A and Grigoletto E (1990) Combined radiotherapy and chemotherapy versus radiotherapy alone in locally advanced epidermoid bronchogenic carcinoma. *Cancer* **65**: 400–404

Trovo MG, Minatel E, Franchin G, Boccieri MG, Nascimben O, Bolzicco G, Pizzi G, Torretta A, Veronesi A, Gobitti C, Zanelli DJ and Monfardini S (1992) Radiotherapy versus radiotherapy enhanced by cisplatin in stage III non-small cell lung cancer. *Int J Radiat Oncol Biol Phys* **24**: 11–15

Wingo PA, Tong T and Bolden S (1995) Cancer statistics, 1995. *CA Cancer J Clin* **45**: 8–30

World Health Organization (1979) *WHO Handbook for Reporting Results of Cancer Treatment*. WHO Offset Publications: Geneva

*© Cancer Research Campaign 1999*
